# New Diagnostic and Therapeutic Tools for Thyroid Cancer

**DOI:** 10.1155/2013/312378

**Published:** 2013-11-26

**Authors:** Eleonore Fröhlich, Richard Wahl, Barbara Czarnocka, Leonidas Duntas

**Affiliations:** ^1^Center for Medical Research, Medical University of Graz, 8010 Graz, Austria; ^2^Department of Medicine IV (Diabetology, Endocrinology, Angiology, Nephrology, and Clinical Chemistry), Medical Clinic, 72076 Tuebingen, Germany; ^3^Medical Center of Postgraduate Education, 01-813 Warsaw, Poland; ^4^Endocrine Unit, Evgenidion Hospital, University of Athens Medical School, 11528 Athens, Greece

Thyroid cancer is the commonest endocrine malignancy but represents only 1% of all cancers. Although the prognosis of differentiated thyroid cancer is good, the survival time of anaplastic and of medullary thyroid cancer is still very short. More precise diagnosis with better stratification of patients and identification of risk patients may improve the prognosis of thyroid cancer. Treatment of thyroid cancer is multidisciplinary and involves endocrinologists, nuclear medicine specialists, and surgeons. The spectrum of contributions to this special issue in International Journal of Endocrinology is well reflecting this situation. 

The contributions to this special issue, two reviews and three studies, report mainly on laboratory and imaging techniques to improve diagnosis in thyroid cancer. J. Hannallah et al. emphasize the multimodal approach in diagnosis and managing patients with poorly differentiated thyroid cancer in their review. The identification of poorly differentiated thyroid cancer has been complicated by the lack of clear criteria for diagnosis. A panel of histological, immunohistochemical, and genetic markers and clinical parameters is listed which are typical for this thyroid carcinoma entity. The value of current treatment possibilities is critically discussed. In the paper review by G. Treglia et al. the role of Fluorine-18-Fluorodeoxyglucose positron emission tomography in aggressive subtypes of different thyroid cancer types (Hürthle cell, anaplastic, poorly differentiated, more aggressive histological subtypes of differentiated thyroid cancer, and metastases) is discussed. FDG-PET-positivity in radio-iodine-refractory differentiated thyroid cancer appears to predict more aggressive tumor progression. Data on imaging of medullary thyroid carcinoma lesions using glucagon-like peptide 1 by [Lys40(Ahx-HYNIC-99mTc/EDDA)NH2]-exendin-4 are presented by D. Pach et al. Radioactively labeled GLP-1 analogues are successfully used in patients with insulinoma but the diagnostic value in medullary thyroid cancer is not clear. According to the first preliminary data, the analogues may possess a confirmatory role in lesions where inconsistent results are obtained with other imaging procedures. S. H. Hsieh et al. in their study identified male gender as prognostic parameter for higher recurrence in papillary thyroid cancer in stages II–IV. The study by B. C. Ahn et al., by contrast, deals with a very established clinical test, the measurement of serum thyroglobulin levels, for the identification of recurrent lesions in differentiated thyroid cancer. The importance of patients' antithyroglobulin levels in the calculation of thyroglobulin levels is addressed.

The identification of potentially aggressive subtypes of thyroid cancer and the early detection of recurrent lesions play key roles in the managing of the patient. By compiling these papers, we hope to add some knowledge with respect to screening and postoperative care particularly of the more aggressive thyroid cancer types.

*Eleonore Fröhlich* *Eleonore Fröhlich* *Richard Wahl* *Richard Wahl* *Barbara Czarnocka* *Barbara Czarnocka* *Leonidas Duntas* *Leonidas Duntas*

